# Nano selenium-enriched probiotic *Lactobacillus* enhances alum adjuvanticity and promotes antigen-specific systemic and mucosal immunity

**DOI:** 10.3389/fimmu.2023.1116223

**Published:** 2023-01-27

**Authors:** Runhang Liu, Weijiao Sun, Tianzhi Sun, Wenzhi Zhang, Yongchao Nan, Zheng Zhang, Kongrui Xiang, Hongliang Yang, Fang Wang, Junwei Ge

**Affiliations:** ^1^ College of Veterinary Medicine, Northeast Agricultural University, Harbin, China; ^2^ State Key Laboratory of Veterinary Biotechnology, Harbin Veterinary Research Institute, The Chinese Academy of Agricultural Sciences, Harbin, China; ^3^ Heilongjiang Provincial Key Laboratory of Zoonosis, Harbin, China

**Keywords:** nano selenium-enriched *L. brevis* 23017, alum adjuvants, immunoenhancement, SIgA antibody, selenoprotein

## Abstract

Nano selenium-enriched probiotics have been identified to improve immune responses, such as alleviating inflammation, antioxidant function, treatment of tumors, anticancer activity, and regulating intestinal flora. However, so far, there is little information on improving the immune effect of the vaccine. Here, we prepared nano selenium-enriched *Levilactobacillus brevis* 23017 (SeL) and heat-inactivated nano selenium-enriched *L. brevis* 23017 (HiSeL) and evaluated their immune enhancing functions on the alum-adjuvanted, inactivated *Clostridium perfringens* type A vaccine in mouse and rabbit models, respectively. We found that SeL enhanced immune responses of the vaccine by inducing a more rapid antibody production, eliciting higher immunoglobulin G (IgG) antibody titers, improving secretory immunoglobulin A (SIgA) antibody level and cellular immune response, and regulating Th1/Th2 immune response, thus helping to induce better protective efficacy after challenge. Moreover, we confirmed that the immunoenhancement effects are related to regulating oxidative stress, cytokine secretion, and selenoprotein expression. Meanwhile, similar effects were observed in HiSeL. In addition, they show enhanced humoral immune response at 1/2 and 1/4 standard vaccine doses, which confirms their prominent immune enhancement effect. Finally, the effect of improving vaccine immune responses was further confirmed in rabbits, which shows that SeL stimulates the production of IgG antibodies, generates α toxin–neutralizing antibodies rapidly, and reduces the pathological damage to intestine tissue. Our study demonstrates that nano selenium-enriched probiotics improve the immune effect of the alum adjuvants vaccine and highlight its potential usage in remedying the disadvantages of alum adjuvants.

## 1 Introduction

Nano selenium-enriched probiotics can be considered a new form of Se organic products (1). The preparation of nano selenium-enriched probiotics adopts a green, safe, efficient, and low-cost biological transformation method. Briefly, sodium selenite was added into the growth medium of probiotics, and the reductase of probiotics converts inorganic sodium selenite to predominately nano selenium, which is then transformed into nano selenium-enriched probiotics (2–4). Nano selenium-enriched probiotics increased immune responses, enhanced antioxidant function (5), and alleviated inflammation-induced intestinal injury (6). In addition, nano selenium-enriched probiotics significantly inhibited tumor growth (7), increased the lifespan of breast cancer-bearing mice (8), regulated intestinal flora, and significantly reduced the number of pathogenic bacteria (9). These research studies have clarified that nano selenium-enriched probiotics play a significant role in the treatment of diseases and immune modulation.

The positive effects of nano selenium-enriched probiotics resulted from the modulation of antioxidant properties and cytokines release. The antioxidant function of nano selenium-enriched probiotics was attributed to significantly improved (10) activities of intracellular antioxidant enzymes, such as thioredoxin reductase, glutathione peroxidase, and glutathione peroxidase reductase. Nano selenium-enriched probiotics reduced the level of inflammatory factors interleukin-1β (IL-1β), IL-6, IL-12p70, IL-17A, IL-21, IL-23, tumor necrosis factor–α (TNF-α), and interferon-γ (IFN-γ) and increased the level of IL-10 (11, 12). Other mechanisms contributed to the restrained expression of nuclear factor-κB (NF-κB) and Toll-like receptor 4 and the increased number of tight junction proteins (occludin, ZO-1, and claudin-1) (13). Furthermore, nano selenium-enriched probiotics improved macrophage function activity to keep beneficial effects (14) and remarkably restrained the reduction of goblet cell numbers (15).

At present, alum adjuvants continue to be commonly used globally. Billions of vaccines containing alum adjuvants have been successfully administered in humans and animals since first use in 1932 and greatly contributed to decreasing the mortality and morbidity of infectious diseases (16). Alum adjuvants improved humoral immune response (17) and also promoted the uptake of antigens by antigen-presenting cells (18). Meanwhile, alum adjuvants stimulated endogenous-cellular immune responses mediated by NLRP3 and encouraged macrophages to secrete high levels of pro-inflammatory factors such as IL-1β and IL-18 (19). In addition, alum adjuvants were widely used due to their comparatively lower cost and outstanding safety. However, there are still some drawbacks of alum adjuvants, which is poor in inducing Th1 immune response (20) and fails to promote mucosal immune response (21) to produce secretory immunoglobulin A (SIgA) antibodies. Considering the disadvantages of alum adjuvants, we put forward an idea of whether nano selenium-enriched probiotics could be used to improve alum adjuvanticity, thus improving the vaccine’s protective effect.

This study aims to prepare nano selenium-enriched *L. brevis* 23017 (SeL) and heat-inactivated nano selenium-enriched *L. brevis* 23017 (HiSeL) and to evaluate whether they can improve the immune effect of the vaccine, which possibly be used as vaccine immune enhancers. For this purpose, (i) SeL and HiSeL were prepared through an optimized process with the advantages of simple, quick, and mild reaction conditions and no need for specific equipment; (ii) they were used to evaluate the impact on the immune efficacy of the vaccine by analyzing humoral immunity, mucosal immunity, Th1/Th2 response, protective efficacy after challenge, and other related indicators in a mouse model; (iii) they were also used to determine the immunopotentiation effect for 1/2 and 1/4 standard vaccine doses; (iv) the mechanisms related to immune enhancement were also sought to be revealed; and (v) SeL was applied to conduct a series of experiments in rabbits, including antibody detection, protective efficacy after challenge, and toxin neutralization reaction. Our results show that SeL can be applied to improve the vaccine effect and possess the potential to remedy the disadvantages of alum adjuvants.

## 2 Materials and methods

### 2.1 Animals and ethics statement

Female Kunming mice (4- to 6-week-old) and rabbits (6-week-old) were purchased from the Laboratory Animal Center of the Second Affiliated Hospital of Harbin Medical University (Harbin, China). The mice and rabbits were maintained under constant conditions (25 ± 1°C and 65% humidity) and had free access to water and food under a 12-h light/dark program. After the mice and rabbits adapted to the laboratory conditions for 1 week, they were randomly divided into groups. The Ethical Committee of the Institute approved all scientific experiments. All applicable international and national guidelines for the care and use of animals in experiments were followed and approved by the Institutional Committee of Northeast Agricultural University (NEAUEC20210326).

### 2.2 Biological synthesis of SeL

*L. brevis* 23017 was obtained from our laboratory (22) and stored in the China Center for Type Culture Collection (CCTCC). Its strain conservation number was CCTCC AB 2018164. The preparation of SeL was carried out as previously described (23) with slight modifications. In short, when the bacterial cultures reached an OD_600nm_ of 0.25, sodium selenite was added to a final concentration of 30 μg/ml, and the bacterial cells were cultured at 37°C for another 18 h. HiSeL was obtained according to the method of Murosaki et al. (24). Transmission electron microscopy (TEM) analysis was assayed following the procedure reported by Raya et al. (25).

### 2.3 Immunization of mice

The rabbit vaccine, alum-adjuvanted, inactivated *Clostridium perfringens* type A vaccine (catalog 151826001) (Lvdu Biotechnology Co., Ltd.), was selected as the model vaccine in this study. After being adapted to the laboratory conditions, the mice were randomly divided into five groups (n = 10 per group) (1): control group: the mice were gavaged with 200 μl of phosphate buffered saline (PBS) once daily for 5 consecutive days on days 1–5 (2); vaccine group: the mice were gavaged with 200 μl of PBS once daily for 5 consecutive days on days 1–5, and then, one standard dose vaccine (200 μl) was subcutaneously injected on day 6 (3); L + Vac group: the mice were gavaged with 200 μl of *L. brevis* 23017 (1 × 10^9^ colony forming units CFU) once daily for 5 consecutive days on days 1–5, and then, 200 μl of vaccine was subcutaneously injected on days 6 (4); SeL + Vac group: the mice were gavaged with 200 μl of SeL (1 × 10^9^ CFU) once daily for 5 consecutive days on days 1–5, and then, 200 μl of vaccine was subcutaneously injected on days 6 (5); HiSeL + Vac group: the mice were gavaged with 200 μl of PBS once daily for 5 consecutive days on days 1–5, and then, 200 μl of vaccine was subcutaneously injected, and hiSeL (1 × 10^6^ CFU) was intraperitoneally injected on day 6. Experiments were carried out for 34 days. After 24 hours of immunization, five mice in each group were randomly selected to detect antioxidant capacity, cytokines level, and selenoprotein expression. Then, after 21 days of immunization, the remaining mice were intraperitoneally challenged with the α toxin of *Clostridium perfringens* type A (C57-8) at a dose of 1 × LD_100_ (70 μl), and the body weight changes, the clinical symptoms, and survival rates were watched and recorded over the next 7 days. Sera and fecal samples were gathered on days 7, 10, 14, and 28 after immunization to detect antibody response. At 28 days after immunization, mice were sacrificed, and the sera, fecal, spleen, jejunal tissue, and intestinal samples were collected for further use.

### 2.4 Immunization of mice with 1/2 and 1/4 standard vaccine doses

To further show whether SeL and HiSeL enhanced the effect of the vaccine, we conducted an evaluation using 1/2 and 1/4 standard vaccine doses (1). In the 1/2 or 1/4 vaccine group, the mice were gavaged with 200 μl of PBS once daily for 5 consecutive days on days 1–5, and then, 100 or 50 μl of vaccine was subcutaneously injected on days 6 (2); in the SeL + 1/2 Vac or SeL + 1/4 Vac group, the mice were gavaged with 200 μl of SeL (1 × 10^9^ CFU) SeL once daily for 5 consecutive days on days 1–5, and then, 100 or 50 μl of vaccine was subcutaneously injected on days 6 (3); in the HiSeL + 1/2 Vac or HiSeL + 1/4 Vac group, prior to immunization, the mice were gavaged with 200 μl of PBS once daily for 5 consecutive days on days 1–5, and then, 100 or 50 μl of vaccine was subcutaneously injected, and HiSeL (1×10^6^ CFU) was intraperitoneally injected on days 6. Afterward, the challenge and sample collection procedures are the same as those for the above standard dose.

### 2.5 Immunization of rabbits

After the rabbits adapted to the laboratory conditions for 1 week, they were randomly divided into three groups (n = 5 per group) (1): control group: the rabbits were gavaged with 5 ml of PBS once daily for 5 consecutive days on days 1–5 (2); vaccine group: the rabbits, were gavaged with 5 ml of PBS once daily for 5 consecutive days on days 1–5, and then, 2 ml of vaccine was subcutaneously injected on days 6 (3); SeL + Vac group: prior to immunization, the rabbits were gavaged with 5 ml of 5 × 10^10^ CFU SeL once daily for 5 consecutive days on days 1–5, and then, 2 ml of vaccine was subcutaneously injected on days 6. Experiments were carried out for 34 days. Then, 21 days after the immunization, the rabbits were intraperitoneally challenged with the α toxin of *Clostridium perfringens* type A at a dose of 1 × LD_100_ (2.5 ml), and the clinical symptoms and survival rates were watched and recorded over the next 7 days. Sera samples were gathered on days 7, 10, 14, and 28. Five rabbits were sacrificed for analysis on days 28 after immunization, and the sera and jejunal tissue samples were harvested for subsequent experiments.

### 2.6 Antibody measurement

α Toxin is the major virulence factor of *Clostridium perfringens* type A, so the antibody against α toxin is the most used indicator to evaluate the vaccine effect (26). Detection of specific immunoglobulin G or SIgA was examined by indirect enzyme-linked immunosorbent assay (ELISA) according to Li et al. (27) with some modifications. In short, vectors pET32a (catalog 69015-3) (Novagen company, USA) were used to construct recombinant α toxin protein expression plasmids. Recombinant α toxin proteins expressed by *E. coli* Rosetta (DE3) (catalog EC1010) (Shanghai Wei Di Biotechnology Co., Ltd.) were purified by Ni-column. In the tests, purified α toxin recombinant protein of approximately 3 μg/ml was used as the coating antigen. Peroxidase-conjugated anti-mouse IgG antibodies (catalog A0216) (1:5,000; Biotechnology Co., Ltd., Shanghai), peroxidase-conjugated anti-mouse IgA antibodies (catalog RS030211) (1:10,000; ImmunoWay Biotechnology Company), or peroxidase-conjugated anti-rabbit IgG antibodies (catalog A0208) (1:5,000; Boster Bioengineering Co., Ltd., Wuhan) were used. Concentrations of total intestinal mucus SIgA were performed using a commercial mouse ELISA kit (catalog D721136) (Shanghai Sangon Biotech. Co., Ltd.).

### 2.7 Cytokine measurement

TNF-α (catalog EK0527), transforming growth factor–β1 (TGF-β1) (catalog EK0515), IL-5 (catalog EK0408), IL-6 (catalog EK0411), and IL-13 (catalog EK0425) were tested by ELISA kits (Boster Bioengineering Co., Ltd., Wuhan) following the manufacturer’s protocol.

### 2.8 Real-time quantitative PCR

Real-time quantitative PCR (RT-qPCR) was used to analyze the expression of IL-4, IL-10, IL-12, IFN-γ, and selenoprotein genes as described previously (28–31). Jejunal tissue samples were collected to measure cytokines, and spleen samples were collected to detect selenoprotein genes. The primers are listed in **Table S1**. RT-qPCR kits (catalog Q204) were purchased from EnzyArtisan Technology Co., Ltd. (Shanghai). The PCR conditions were as follows: initial denaturation for 10 min at 95°C, followed by 45 cycles of 10 s at 95°C, 59°C for 30 s, and elongation at 72°C for 30 s. The β-actin and Hprt were used as housekeeping genes to normalize the relative expression level. The relative mRNA level was calculated with the 2^−ΔΔCt^ method.

### 2.9 Antioxidant enzyme assay

Malondialdehyde (MDA) (catalog A003-1), superoxide dismutase (SOD) (catalog A001-1), glutathione peroxidase (GSH-Px) (catalog A005-1), and total antioxidant capacity (T-AOC) (catalog A015-1) were measured using the Antioxidant Assay Kits (Nanjing Jiancheng Bioengineering Institute) according to the manufacturer’s instructions.

### 2.10 Toxin neutralization assay

The neutralization of the lethal effect of α toxin was determined according to the method of Schoepe et al. (32) with some modifications. In short, we conducted experiments using the sera of the above three groups of rabbits on the 7th, 10th, and 14th days after immunization. Mice were intraperitoneally injected with preincubated mixtures (37°C, 1 h) of 1 × LD_100_ of α toxin (70 μl) and rabbit sera at a volume ratio of 1:10. Survival rate was recorded over a 1-day period.

### 2.11 Histopathological detection

The jejunal tissue samples of mice and rabbits were fixed in 10% paraformaldehyde at room temperature for at least 48 h. The company was commissioned to make and analyze pathological sections (Wuhan Servicebio Technology Co., Ltd., Wuhan, Hubei, China).

### 2.12 Statistical analysis

Statistical analysis was performed using GraphPad Prism 8.0.1 software. The results were shown as the mean ± SD, and the importance of the differences between groups was analyzed using one-way or two-way ANOVA. Statistical significance was showed as *P < 0.05, **P < 0.01, and ***P < 0.001.

## 3 Results

### 3.1 Preparation and characterization of SeL and HiSeL

**Figure 1:**
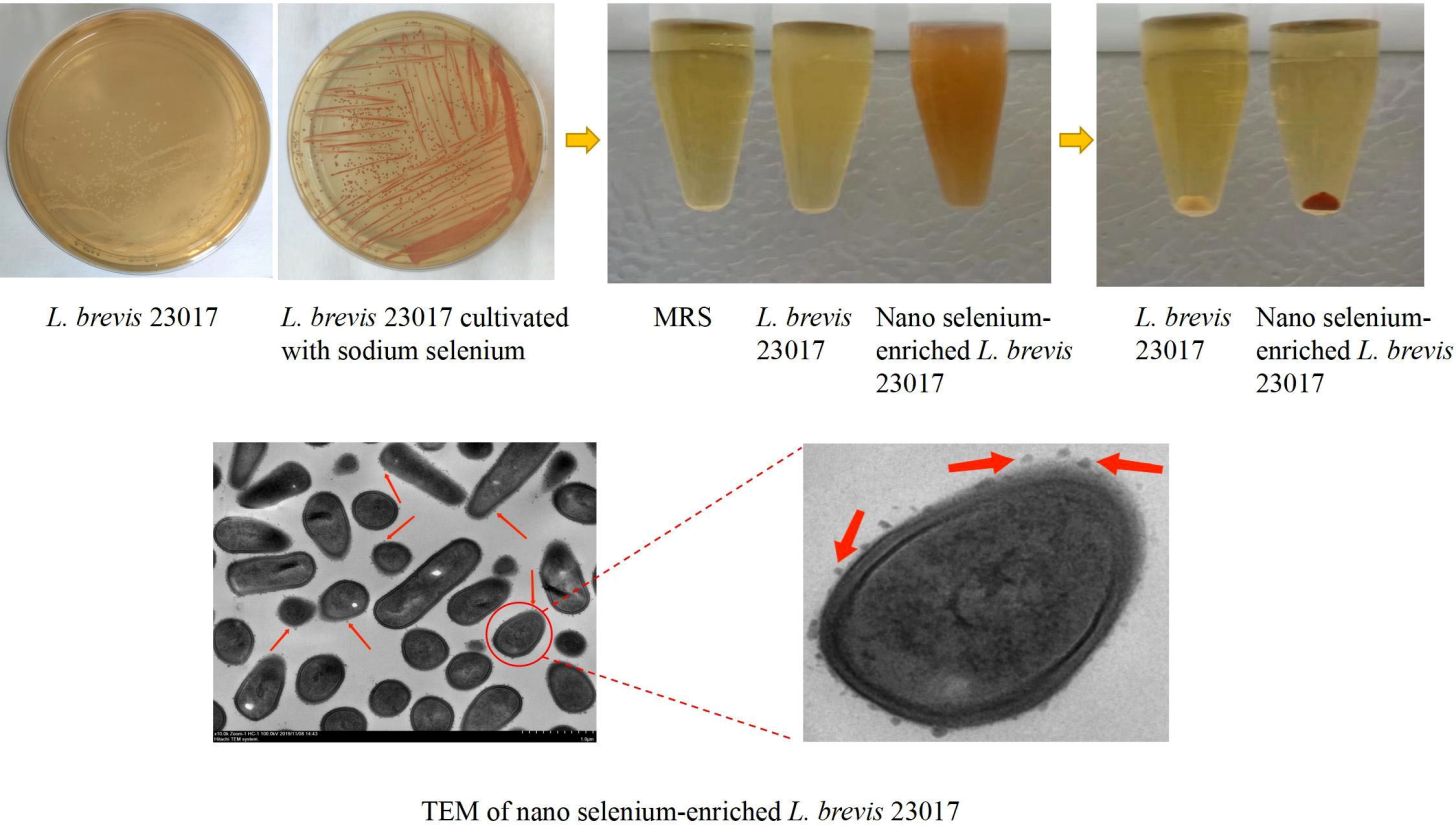
Synthesis and characterization of nano selenium-enriched *L. brevis* 23017. *L. brevis* 23017 cultivated under sodium selenite stress (30 μg/ml) led to reduction of selenite ions (Se^IV^) into extracellular elemental Se (Se^0^) nanoparticles observed by transmission electron microscopy (TEM), and visible as red color. The red arrow in the figure refers to SeNPs.

To prepare SeL more productively, we optimized the reaction conditions, approximately 78.4% transformation efficiency can be achieved under these conditions. As shown in **Figure 1**, the single colony of SeL appeared red when cultured on the MRS agarose medium containing sodium selenium (30 μg/ml). Compared with blank MRS and MRS containing SeL, SeL showed obvious red after being cultured in MRS containing sodium selenium (30 μg/ml) for 18 hours. TEM analysis showed that SeL contained numerous selenium nanoparticles (SeNPs) with approximately 50–80 nm.

### 3.2 SeL and HiSeL delayed the weight loss of mice

To assess the influence of SeL and HiSeL on body weight for immunized mice after the challenge, we recorded weight changes of mice after challenge during 7 days. As shown in **Figure S1**, the weight of mice in the vaccine group had been falling on days 0–4 after challenge and then leveled off on days 4–6 after challenge, whereas the weight of mice in the L + Vac, SeL + Vac, and HiSeL + Vac groups first decreased on days 0–4 after challenge and then increased on days 4–6 after challenge. Moreover, the weight of mice in the non-challenge control group increased steadily. On days 0–4 after challenge, the weight of mice in the L + Vac, SeL + Vac, and HiSeL + Vac groups descended more slowly than the vaccine group. Weight loss was the least in the oral SeL group.

On days 4–6 after challenge, compared with the vaccine group, the weight of mice in the L + Vac, SeL + Vac, and HiSeL + Vac groups gradually increased. However, the mice immunized with the vaccine showed leveling body weight change. Meanwhile, the mice in the SeL + Vac group gained significantly more weight than the L + Vac group. We found that mice in the SeL + Vac group and HiSeL + Vac group show obvious advantages in slowing down weight loss and accelerating weight recovery, especially SeL. Thus, SeL and HiSeL helped improve vaccine effectiveness and played an essential role in regulating body weight after vaccination.

### 3.3 SeL and HiSeL enhanced antigen-specific humoral immunity in mice

**Figure 2:**
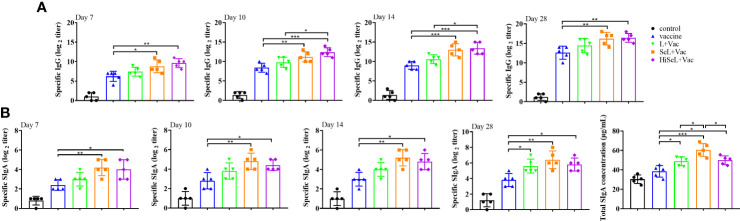
Effect of SeL and HiSeL on the humoral immune response and mucosal immune response induced by the immunization of mice. Sera and fecal samples of mice were gathered on days 7, 10, 14, and 28 after immunization to detect specific IgG and SIgA antibody response. Intestinal mucus samples of mice were gathered on day 28 after immunization to detect total SIgA concentration. **(A)** The titer of specific IgG antibody in mice sera. **(B)** The titer of specific SIgA antibodies in mice fecal and total SIgA concentration in intestinal mucus. Each experimental group consisted of five mice per group. Data were presented as means ± SD (*P < 0.05, and **P < 0.01). The statistical analysis was performed using one-way ANOVA.

To detect the humoral immune level in mice changed, we detected antigen-specific IgG antibodies at days 7, 10, 14, and 28 after immunization. As shown in **Figure 2A**, the IgG level in the control group was unchanged, and IgG antibody levels in other groups improved steadily. On day 7 after immunization, compared with the vaccine group, the IgG content in the sera of the L + Vac group was higher, and the SeL + Vac group (*P <* 0.05) and HiSeL + Vac group (*P <* 0.01) were remarkably higher. This finding suggested that SeL and HiSeL induced the vaccine to produce antibody faster.

On day 10 after immunization, the mice in the SeL + Vac group (*P <* 0.01) or HiSeL + Vac group (*P <* 0.001) had higher levels of antigen-specific IgG than those immunized with vaccine alone, and HiSeL + Vac group (*P* < 0.05) produced more antibodies when compared with L + Vac group. Meanwhile, we observed similar trends on day 14 after immunization.

After the challenge (day 28 after immunization), the level of sera IgG antibody of mice in the SeL + Vac group (*P <* 0.01) or HiSeL + Vac group (*P <* 0.01) was higher than vaccine group, but there is no significant difference between the L + Vac group and vaccine group. These data showed that SeL and HiSeL were remarkably better than *L. brevis* 23017 in enhancing humoral immunity after vaccination.

### 3.4 SeL and HiSeL enhanced antigen-specific mucosal immunity in mice

To detect whether the mucosal immune level of mice has changed, we detected specific SIgA and total intestinal mucus SIgA antibody levels in the intestine. As shown in **Figure 2B**, the antibody titer of specific SIgA and total SIgA in other groups rose gradually except for the control group. On days 7, 10, and 14 after immunization, the SIgA content of mice in the SeL + Vac group (*P <* 0.01) and the HiSeL + Vac group (*P <* 0.05) was remarkably increased when compared with the vaccine group. Meanwhile, the SIgA content of mice in the SeL + Vac group and the HiSeL + Vac group was higher than in the L + Vac group, but there was no significant difference between them. After the challenge (day 28 after immunization), the mice in the L + Vac group (*P <* 0.05), the SeL + Vac group (*P <* 0.01), and the HiSeL + Vac (*P <* 0.05) group secreted remarkably more antigen-specific SIgA antibodies than vaccine group.

The total intestinal mucus SIgA was detected in mice at day 28 after immunization, consistent with the above data. The difference is the SeL + Vac group produced a total SIgA antibody than the L + Vac group (*P <* 0.05) and the HiSeL + Vac group (*P <* 0.05). Collectively, SeL and HiSeL significantly improved mucosal immune response.

### 3.5 SeL and HiSeL enhanced the secretion of cytokines related to SIgA

**Figure 3:**
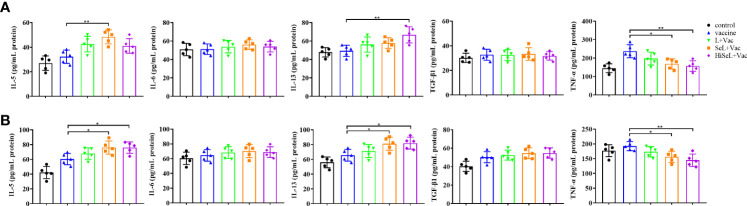
Effect of SeL and HiSeL on the secretion of cytokines related to SIgA induced by the immunization of mice. Sera samples of mice were gathered on days 1 and 28 after immunization to detect cytokines related to SIgA. **(A)** Results of cytokine in sera on the first day after immunization. **(B)** Results of cytokine in sera on the 28th day after immunization. Each experimental group consisted of five mice per group. Data were presented as means ± SD (*P < 0.05, and **P < 0.01). The statistical analysis was performed using one-way ANOVA.

To assess the changes of inflammatory-related factors in sera, we detected IL-5, IL-6, IL-13, TGF-β1, and TNF-α related to SIgA by ELISA. As shown in **Figure 3A**, on day 1 after immunization, in comparison with the control group, the level of IL-5 and IL-13 in other groups was significantly increased, whereas the level of TNF-α showed the opposite trend. It can be seen that the level of IL-5 in the SeL + Vac group (*P* < 0.01) was higher than that in the vaccine group. Similarly, the level of IL-13 in the HiSeL + Vac group (*P* < 0.01) was significantly increased compared with the vaccine group. However, in the vaccine group, the TNF-α level was remarkably higher than that in the SeL + Vac group (*P* < 0.05) and the HiSeL + Vac group (*P* < 0.01).

On day 28 after immunization, as shown in **Figure 3B**, the level of TNF-α in all groups was lower than that in the control group except for the vaccine group. Then, compared with the vaccine group, the level of IL-5 and IL-13 was increased in the L + Vac group (*P <* 0.05), the SeL + Vac group (*P <* 0.05), and the HiSeL + Vac group (*P <* 0.05). Moreover, the levels of IL-5 and IL-13 in the SeL + Vac group and the HiSeL + Vac group increased slightly more than that in the L + Vac group. We also noticed no statistical difference in IL-6 and TGF-β1 at the protein level among all five groups at days 1 and 28 after immunization. Therefore, SeL and HiSeL may associate with cytokine secretion.

### 3.6 SeL and HiSeL regulate the expression of cytokine genes related to SIgA

**Figure 4:**
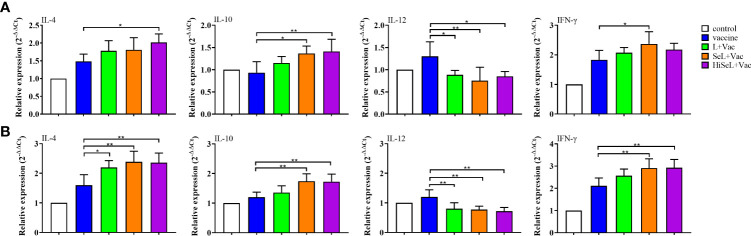
Effect of SeL and HiSeL on the expression of cytokine genes related to SIgA induced by the immunization of mice. Jejunal tissue samples of mice were gathered on days 1 and 28 after immunization to detect cytokine genes related to SIgA. **(A)** Results of mRNA level of cytokines in jejunal cells on the first day after immunization. **(B)** Results of mRNA level of cytokines in jejunal cells on the 28th day after immunization. Each experimental group consisted of five mice per group. Data were presented as means ± SD (*P < 0.05, and **P < 0.01). The statistical analysis was performed using one-way ANOVA.

To determine the changes in inflammatory-related factors in jejunal cells, we detected the mRNA expression of IL-4, IL-10, IL-12, and IFN-γ. As shown in **Figure 4A**, on the first day after immunization, the mRNA expression of IL-12 was significantly decreased, whereas the expression of IL-4, IL-10, and IFN-γ was increased in four experimental groups. IL-4 in the HiSeL + Vac group (*P* < 0.05) was higher than in the vaccine group. Similarly, IL-10 in the SeL + Vac group (*P* < 0.05) and the HiSeL + Vac group (*P* < 0.01) was significantly increased compared with the vaccine group. Compared with the SeL + Vac group (*P* < 0.05), the IFN-γ in the vaccine group declined. However, IL-12 in the vaccine group was significantly (*P* < 0.05) higher than that in other treatment groups.

On the 28th day after immunization (**Figure 4B**), IL-12 in all groups was lower than in the control group except for the vaccine group. Meanwhile, IL-4, IL-10, and IFN-γ were increased in the L + Vac group, the SeL + Vac group (*P <* 0.01), and the HiSeL + Vac group (*P <* 0.01) when compared with the vaccine group. In addition, IL-4, IL-10, and IFN-γ in the SeL + Vac group and the HiSeL + Vac group improved mildly more than in the L + Vac group. There was no significant difference between the SeL + Vac group and the HiSeL + Vac group among all studied cytokine mRNA expression. This finding may further suggest that SeL and HiSeL inspired the creation of different cytokines.

### 3.7 SeL and HiSeL improved the antioxidant function

To measure the levels of oxidative stress in immunized mice, we detected the antioxidant function of the mice sera and jejunal tissue samples on the first and 28th days after immunization. The test results of sera and jejunal tissue are shown in **Table 1**. On the first day after immunization, sera test results showed that compared with the vaccine group (*P <* 0.05) and the L + Vac group (*P <* 0.05), MDA decreased in the SeL + Vac group. SOD in the SeL + Vac group (*P* < 0.01) and the HiSeL + Vac group (*P* < 0.01) was higher than in the L + Vac group and the vaccine group. Similarly, T-AOC in the L + Vac group (*P* < 0.05) and the SeL + Vac group (*P* < 0.05) was significantly increased compared with the vaccine group. Then, jejunal tissue test results showed that MDA and SOD indexes of the SeL + Vac group (*P* < 0.05) revealed notable antioxidant advantage over all groups. Compared with the L + Vac group, the content of GSH-Px in the SeL + Vac group and the HiSeL + Vac group remarkably increased. T-AOC in the L + Vac group (*P* < 0.05), the SeL + Vac group (*P* < 0.01), and the HiSeL + Vac group (*P* < 0.01) were higher than those immunized with the vaccine alone. These data showed that SeL and HiSeL improved the antioxidant function among the indexes examined.

**Table 1:** Antioxidant indices in sera and jejunal tissue results on the first and 28th days after immunization.

Group	MDA (nmol/mgprot)	SOD (U/mgprot)	GSH-Px (U/mgprot)	T-AOC (U/mgprot)
Sera results on the first day after immunization				
Control	7.01 ± 0.29	90.11 ± 2.11	427.34 ± 4.67	2.21 ± 0.10
Vaccine	7.06 ± 0.25	82.83 ± 5.95	440.38 ± 11.20	2.25 ± 0.15
L + Vac	6.75 ± 0.26	98.01 ± 3.28^b*^	458.96 ± 19.22	3.29 ± 0.3^ab*^
SeL + Vac	5.11 ± 1.46^abc*^	120.44 ± 7.93^abc**^	479.87 ± 6.02	3.13 ± 0.17^abc*^
HiSeL + Vac	6.01 ± 0.30	121.73 ± 2.27^abc**^	434.94 ± 13.00	2.96 ± 0.05
Jejunal tissue results on the first day after immunization				
Control	3.61 ± 0.11	31.7 ± 2.62	352.72 ± 11.56	0.71 ± 0.05
Vaccine	4.69 ± 0.62	30.8 ± 1.54	372.16 ± 2.95	0.62 ± 0.10
L + Vac	2.25 ± 1.01	33.9 ± 3.25	331.75 ± 6.09	0.99 ± 0.143^ab*^
SeL + Vac	1.80 ± 0.39^b*^	37.6 ± 3.22^ab*^	433.27 ± 6.59^ac*^	1.16 ± 0.20^ab**^
HiSeL + Vac	2.60 ± 0.66	35.9 ± 4.46	427.38 ± 5.82^ac*^	1.08 ± 0.08^ab**^
Sera results on the 28th day after immunization				
Control	7.18 ± 0.16	98.64 ± 5.82	452.34 ± 1.82	1.87 ± 0.43
Vaccine	6.84 ± 0.13	90.92 ± 2.96	449.43 ± 4.58	1.25 ± 0.18^a*^
L + Vac	6.62 ± 0.44	95.01 ± 1.54	483.39 ± 13.83^b*^	1.44 ± 0.61
SeL + Vac	6.87 ± 1.55	97.09 ± 3.44	477.07 ± 23.33	1.71 ± 0.49
HiSeL + Vac	6.54 ± 0.91	92.79 ± 6.27	454.94 ± 10.54	1.54 ± 0.21
Jejunal tissue results on the 28th day after immunization				
Control	3.12 ± 0.82	29.97 ± 0.46	323.72 ± 10.56	0.93 ± 0.14
Vaccine	4.31 ± 1.21	25.77 ± 1.39	272.16 ± 5.86^a*^	0.77 ± 0.12
L + Vac	5.11 ± 0.60^ab*^	29.24 ± 1.43	337.20 ± 14.82	0.90 ± 0.06
SeL + Vac	3.84 ± 0.86	26.98 ± 1.33	344.91 ± 6.59^b*^	0.83 ± 0.08
HiSeL + Vac	3.39 ± 0.26	27.29 ± 1.7	318.03 ± 7.86	1.02 ± 0.13^b*^

Sera and jejunal tissue samples of mice were gathered on days 1 and 28 after immunization to detect antioxidant function. ^a^vaccine/L + Vac/SeL + Vac/HiSeL + Vac group versus control group, ^b^L + Vac/SeL + Vac/HiSeL + Vac group versus vaccine group. ^c^SeL + Vac/HiSeL + Vac group versus L + Vac group. Each experimental group consisted of five mice per group. Data were presented as means ± SD (*P < 0.05, and **P < 0.01). The statistical analysis was performed using one-way ANOVA.

On the 28th day after immunization, sera test results showed that only the L + Vac group revealed significantly higher GSH-Px content than the vaccine group. Then, jejunal tissue test results showed that the content of MDA increased in the L + Vac group (*P <* 0.05). Meanwhile, the content of GSH-Px in the SeL + Vac group (*P* < 0.05) was higher than in those immunized with the vaccine alone. The content of T-AOC in the HiSeL + Vac group (*P* < 0.05) was remarkably increased compared with the vaccine group. However, there was no statistical difference in the SOD index among these groups. The difference in antioxidant function between the groups was reduced, and the redox status of mice was relatively balanced at the 28th day after immunization. These results suggested that SeL and HiSeL displayed competent antioxidant functions.

### 3.8 SeL and HiSeL enhanced the expression of selenoprotein

**Figure 5:**
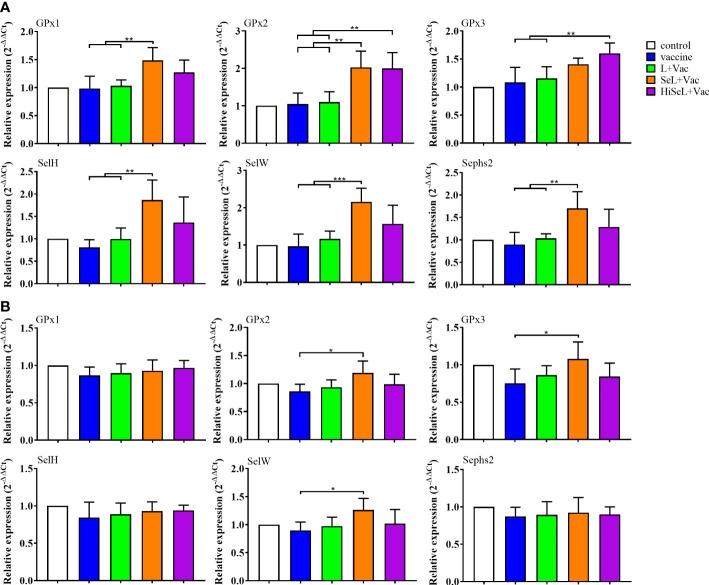
Effect of SeL and HiSeL on the mRNA levels of selenoprotein-related functional genes induced by the immunization of mice. Spleen samples of mice were gathered on days 1 and 28 after immunization to detect selenoprotein-related functional genes. **(A)** Results of mRNA level of selenoprotein-related functional genes in spleen on the first day after immunization. **(B)** Results of mRNA level of selenoprotein-related functional genes in spleen on the 28th day after immunization. Each experimental group consisted of five mice per group. Data were presented as means ± SD (*P < 0.05, **P < 0.01, and ***P < 0.001). The statistical analysis was performed using one-way ANOVA.

To measure the effect of SeL and HiSeL on the expression level of selenoprotein in immunized mice, we randomly dissected five mice in each group on the first day after immunization, collected the spleen of mice, and detected the mRNA expression level of 25 selenoprotein-related genes. As shown in **Figure 5A**, we revealed six representative images with a significant difference. Other genes with no statistical differences were not shown, such as GPx4, SelI, SelK, SelT, SelO, Sepp1, Sep15, and Dio1. Afterward, the expression of GPx1, GPx2, SelH, and Sephs2 genes in the SeL + Vac group (*P <* 0.05) was higher than that in the vaccine group and the L + Vac group. Compared with the vaccine group and the L + Vac group, the expression of the SelW gene in the SeL + Vac group (*P <* 0.01) increased remarkably. Similarly, the expression of GPx2 and GPx3 genes in the HiSeL + Vac group (*P <* 0.05) increased significantly compared with that in the vaccine group and the L + Vac group. GPx1, GPx2, SelH, Sephs2, and SelW genes increased significantly in the SeL + Vac group, and in the HiSeL + Vac group, the expression of GPx2 and GPx3 genes was increased.

Selenoprotein expression was also determined at day 28 after immunization (**Figure 5B**). We only showed representative data, including GPx1, GPx2, GPx3, SelH, SelW, and Sephs2 genes. In the SeL + Vac group (P < 0.05), the expression of GPx2, GPx3, and SelW was conspicuously higher than in the vaccine group. Moreover, all other groups did not differ significantly because the body consumed most selenoproteins on the 28th day after immunization. However, GPx2, GPx3, and SelW genes maintained a competent level, which may imply that these genes were closely related to immune regulation. The results showed that SeL and HiSeL increased the expression of selenoprotein.

### 3.9 SeL and HiSeL enhanced the production of neutralizing antibody

To evaluate whether SeL and HiSeL improve the production of neutralizing antibodies of vaccine, we conducted a protective efficacy against α toxin of *Clostridium perfringens* type A challenge experiment. As shown in **Table S2**, after the mice were injected intraperitoneally α toxin of *Clostridium perfringens* type A, the morbidity rate of the mice in the vaccine group reached 80%, and the mortality rate was 0%. These data may suggest that vaccines could not provide enough neutralizing antibodies for mice. However, the morbidity and mortality rates of the mice in the SeL + Vac group and the HiSeL + Vac group were 0%. By differentiating the clinical observations between the groups, we found that SeL and HiSeL improved the vaccine’s protection, indicating that SeL and HiSeL enhanced neutralizing antibodies of vaccination.

### 3.10 SeL and HiSeL helped to alleviate tissue damage in mice

**Figure 6:**
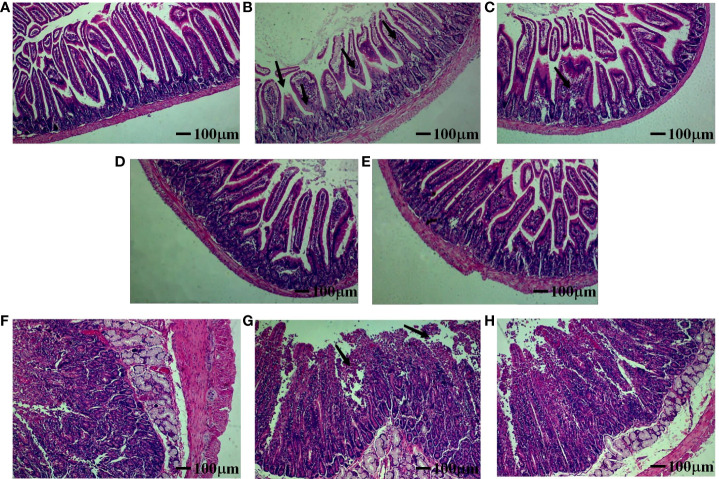
Effect of SeL or HiSeL on the pathological changes of jejunal tissue induced by the immunization of mice and rabbits after challenge. Jejunal tissue samples of mice and rabbits were gathered on day 28 after immunization to detect pathological changes. The pathological changes were examined by HE staining (magnification of ×40), the black arrows point at the lesion location. **(A)** Control group of mice. **(B)** Vaccine group of mice. **(C)** L + Vac group of mice. **(D)** SeL + Vac group of mice. **(E)** HiSeL + Vac group of mice. **(F)** Control group of rabbits. **(G)** Vaccine group of rabbits. **(H)** SeL + Vac group of rabbits.

To determine whether jejunal tissue was affected by α toxin infection, we prepared pathological sections to observe the intestinal injury. As shown in **Figure 6**, in the control group (**Figure 6A**), the jejunal tissue of mice had no obvious pathological changes. Only the vaccine group (**Figure 6B**) showed apparent pathological changes with the disordered arrangement and slight intestinal villi damage, and the intestinal villi gap was also enlarged, the number of goblet cells increased in the mucosal layer, and inflammatory cells appeared to infiltrate. In the L + Vac group (**Figure 6C**), there was a mild injury in jejunal tissue, such as an enlarged intestinal villi gap and increased inflammatory cells.

Tissues from the SeL + Vac group (**Figure 6D**) and the HiSeL + Vac group (**Figure 6E**) did not exhibit significant tissue changes. The morphology and structure of jejunal tissue were roughly normal. Meanwhile, the intestinal mucosa was intact, the villi of the intestine were well-arranged, and staining was more uniform in both the cytoplasm and nucleus. Lamina propria inflammatory cell infiltrates lightly were also observed. Thus, SeL and HiSeL were able to help immunized mice strengthen immunity and reduce intestinal pathological changes.

### 3.11 SeL and HiSeL enhanced antigen-specific humoral immunity and protective efficacy after challenge at low-dose vaccine in mice

**Figure 7:**
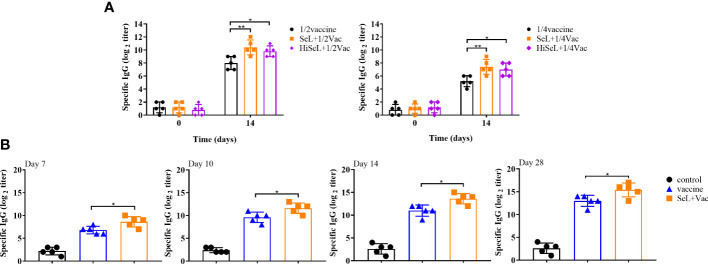
Effect of SeL or HiSeL on the humoral immune response induced by the immunization of mice and rabbits. Sera samples of mice were gathered on days 0 and14 after immunization with 1/2 and 1/4 standard vaccine doses to detect specific IgG antibody response. Sera samples of rabbits were gathered on days 7, 10, 14, and 28 after immunization to detect specific IgG antibody response. **(A)** The titer of specific IgG antibody in mice sera immunized with 1/2 and 1/4 standard vaccine doses. **(B)** The titer of specific IgG antibody in rabbit sera. Each experimental group consisted of five mice or rabbits per group. Data were presented as means ± SD (*P < 0.05, and **P < 0.01). The statistical analysis was performed using one-way ANOVA or two-way ANOVA.

To show the immune enhancement effect of 1/2 and 1/4 standard vaccine doses, we detected IgG antibody levels and the effect of defense against α toxin. As shown in **Figure 7A**, there was no difference in IgG antibody levels among all groups on day 0 after immunization. On day 14 after immunization, the groups with SeL and HiSeL showed higher antibody levels than the vaccine group. Meanwhile, the IgG antibodies in all groups improved steadily with increased time. Mice in the SeL + 1/2Vac group (*P <* 0.01) and the HiSeL + 1/2Vac group (*P <* 0.05) produced additional IgG antibodies when compared with the 1/2vaccine group. Similarly, in comparison with the 1/4vaccine group, mice in the SeL + 1/4Vac group (*P <* 0.01) and the HiSeL + 1/4Vac group (*P <* 0.05) showed a better IgG antibody level. SeL and HiSeL also increased IgG antibody titers when the vaccine was 1/2 and 1/4 standard doses, which may further suggest they improve the humoral immune response in mice.

As shown in **Table S2**, after the challenge, the morbidity rate of the mice in the SeL + 1/2 Vac group and the SeL + 1/4 Vac group was 20%, whereas the morbidity rate of the 1/2 vaccine group and 1/4 vaccine group reached 100%. SeL provided 80% protective efficacy after the challenge for mice at low-dose vaccine, which further indicated SeL boosted the immune enhancement effect of the vaccine. Moreover, the mortality rate was 0% in the SeL + 1/2 Vac group and the SeL + 1/4 Vac group. It can be seen that the survival rate in the HiSeL + 1/2 Vac group and the HiSeL + 1/4 Vac group reached 80% and 40%, respectively, better than that in the vaccine group. On the basis of these findings, SeL and HiSeL strengthened the defense effect of the vaccine.

### 3.12 SeL enhanced antigen-specific humoral immunity and defense against α toxin in rabbits

The above mouse experiments were analyzed, and we selected SeL, which revealed the best results for subsequent rabbit experiments. We detected antigen-specific IgG antibody levels and analyzed the DAI score to evaluate whether SeL improved humoral immune levels and protective efficacy after the vaccine challenge in rabbits. As shown in **Figure 7B**, on days 7, 10, and 14 after immunization, the level of IgG in the control group was unchanged, and the level of IgG in the vaccine group and SeL + Vac group increased steadily. The IgG level in the SeL + Vac group (*P* < 0.05) increased significantly compared with that in the vaccine group. After the challenge (on day 28 after immunization), the mice in the SeL + Vac group (*P <* 0.05) also obviously showed higher IgG antibody levels than those immunized with vaccine alone, which indicated that SeL notably enhanced humoral immune response.

**Figure 8:**
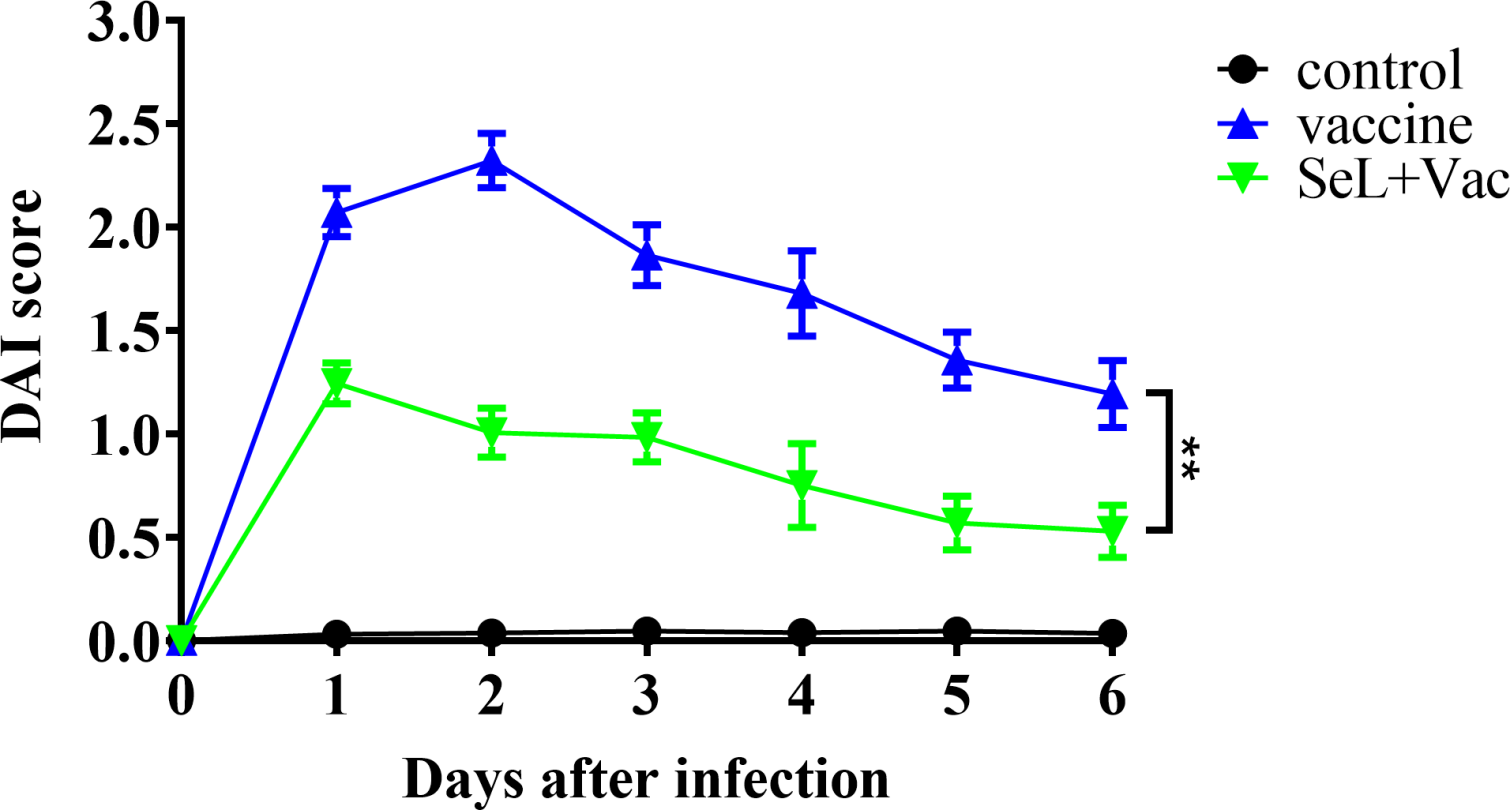
Effect of SeL on the clinical symptoms of immunized rabbits after challenge. The clinical symptoms of rabbits were watched and recorded on days 0–6 after challenge. The results were expressed as DAI score. Each experimental group consisted of five rabbits per group. Data were presented as means ± SD (**P < 0.01). The statistical analysis was performed using two-way ANOVA.

As shown in **Figure 8**, on days 0–6 after challenge, the rabbits in the non-challenge control group had the lowest DAI score and appeared healthy. DAI score curves for the vaccine group and the SeL + Vac group exhibited an initial upregulation followed by a decreasing tendency. The difference is that the SeL + Vac group declined 1 day earlier than the vaccine group because the rabbits administered orally with SeL recovered faster after the challenge. Moreover, the rabbits in the vaccine group had anorexia, depression, curling, hematochezia, weight loss, and other abnormal behaviors, whereas the clinical signs were mild in the SeL + Vac group, such as depression and weight loss slightly. Indeed, SeL strengthened immunity in rabbits against α toxin.

### 3.13 SeL improved the neutralizing antibody produced by immunized rabbits

In the neutralization test (**Table S3**), there was no neutralizing antibody in the sera of the control group rabbits and the sera at seventh day after immunization in the vaccine group and the SeL + Vac group. However, in the sera of the vaccine group and SeL + Vac group on the 10th day after immunization, the survival rate reached 66% and 100%, respectively. The SeL + Vac group shows a better protective effect than the vaccine group. These findings showed that SeL promoted the vaccine to produce neutralizing antibodies faster and provide better protection.

### 3.14 SeL assisted to mitigate tissue injury in rabbits

To evaluate the pathogenicity of α toxin in rabbits, histopathological analyses were performed on the jejunal tissue of rabbits infected with α toxin. The cell structure was normal, and staining was uniform in the cytoplasm and the nucleus (**Figure 6F**). No inflammatory cellular infiltrates were observed. However, in the vaccine group (**Figure 6G**), the arrangement of intestinal villi was disordered. There was necrosis or disintegration in a few intestinal epithelial cells, and interstitial spaces were slightly enlarged. Then, the intestinal villus of rabbits in the SeL + Vac group (**Figure 6H**) was relatively complete, and no shedding of villous epithelial cells. A few inflammatory cell infiltrates were observed in the connective tissue of lamina propria and submucosa. On the basis of these findings, we indicated that SeL effectively helped reduce pathological damage of immunized rabbits after the challenge.

## 4 Discussion

The beneficial effects of nano selenium-enriched probiotics on health and nutrition have been reported (33, 34). Previous studies also highlighted the roles of nano selenium-enriched probiotics in treating diseases in humans and animals (35, 36). However, whether nano selenium-enriched probiotics can be used to improve the immune effect of the vaccine has yet to be studied. Here, we first explore its feasibility and efficacy as a vaccine immune enhancer from different aspects of immune response by conducting experiments in mice and rabbits. We found that SeL and HiSeL improve immune responses to the alum-adjuvanted vaccine, showing faster antibody production, higher antigen-specific IgG and SIgA antibody titers, and stronger cellular immune responses, with better protective efficacy after challenge, which are related to regulating oxidative stress, cytokine secretion, and selenoprotein expression.

SeL successfully improved the effect of the alum-adjuvanted vaccine in the active or heat-inactivated state. Previous studies have shown that lactic acid bacteria improve the immune effect of vaccines (37, 38). Similarly, Se also showed an important role in enhancing the immune effect of the live bivalent vaccine of infectious bronchitis virus and Newcastle disease virus in chickens and attenuated pseudorabies virus vaccine (39, 40). Lactic acid bacteria and Se display synergistic effects in the present study. In the mouse model, after adding SeL, a significant increment in specific antibody levels was found only 7 day after the immunization. SeL effectively stimulates host immunity, especially mucosal immunity. SeL also significantly enhanced the cytokine levels, such as IL-5 and IFN-γ, and then reduced IL-12 production.

Meanwhile, immune responses in the HiSeL-treated mice were examined. HiSeL also promoted faster production of antibodies, and it tended to stimulate the humoral immune response. In terms of cytokines, HiSeL conspicuously reduced the level of TNF-α while increasing the production of IL-4, IL-10, and IL-13. Moreover, both SeL and HiSeL helped the vaccine delay the weight loss of mice after the challenge and reduce the pathological damage of intestine tissue. After the challenge, the vaccine showed an efficacy of 100% protection for immunized mice due to incorporating SeL or HiSeL. Unexpectedly, both showed enhanced IgG antibody levels and helped the vaccine provide high protection efficiency for mice when immunized mice with 1/2 and 1/4 standard vaccine doses. In rabbit experiments, we found a significant difference between immunized mice with SeL and vaccine and immunized mice with vaccine alone only on day 7 after immunization due to SeL inducing more rapid humoral immune responses. DAI score was recorded on days 0–6 after challenge, the mice immunized with SeL and vaccine showed lower scores than those immunized with vaccine alone. The data indicated that SeL improves defense against α toxin of the vaccine. In the toxin neutralization assay, the survival rates of the mice immunized with the vaccine reached 66%, whereas the mice immunized with SeL and vaccine reached 100%. Thus, SeL promoted the body to produce more neutralizing antibodies. As shown, SeL and HiSeL should be used as promising immune enhancers to improve the immune effect of the alum-adjuvanted vaccine, and HiSeL has a notable effect at primary immunization and is amenable to long-term storage.

SeL and HiSeL improved the levels of specific SIgA and total SIgA antibodies, effectively making up for alum adjuvants’ natural disadvantage. Many studies have shown that alum adjuvants effectively produce Th2 responses and antigen-specific IgG, but they cannot induce good mucosal immune responses (41, 42). SIgA, the most abundant antibody isotype within the body, is the first line of defense against pathogens and harmful substances in the mucosal immune system (43, 44). Our data show that lactic acid bacteria increased the level of the SIgA antibody, which is consistent with that by Kusumo et al. (45). The level of antigen-specific SIgA antibody in the SeL group and HiSeL group had a better advancement effect than L + Vac group, which suggested SeL and HiSeL promoted to produce more specific SIgA antibody and showed an enhanced mucosal immune response. In addition, we also observed that SeL and HiSeL stimulated the body to secrete more intestinal mucus SIgA than the mice immunized with *L. brevis* 23017 and vaccine, and the effect of SeL was the greatest among them. Subsequently, the cytokine related to SIgA was detected, and we found that increasing the level of SIgA is closely related to an increase in IL-4, IL-5, and IL-10, which is consistent with the study of Beagley and Wu et al. (46, 47). Then, Wu et al. (48) found that IL-13 has a similar effect, which is consistent with our results.

Moreover, previous studies found that differentiating B cells into plasma cells secreting IgA occurs upon interactions with T-cells in the lamina propria in an environment rich in IL-4 and other Th2 cytokines (49). Another study showed that IFN-γ and IL-4 promoted the passage of IgA through intestinal epithelium and increased IgA transmembrane transport (50). From this, the increment of SIgA antibody may be due to increased Th2-type cytokine (IL-4, IL-5, IL-10, and IL-13) levels. However, Th1-type cytokine (IFN-γ) levels were also increased, suggesting that Th2-type cytokines are major influence factors for SIgA antibodies and relate to Th1-type cytokines.

Notably, SeL and HiSeL elicited higher antibody titers and promoted antibody production in advance. Inducing faster vaccine protection is very useful for the body because it will shorten the time between vaccination and exposure, especially during the pandemic or epidemic outbreaks (51). Previous studies have shown that lactic acid bacteria increased the IgG antibody (52) and the SIgA antibody (53), which is consistent with our research. Similarly, Khattab et al. (54) found that selenium-enriched lactic acid bacteria improved the level of IgG in the body. In the present study, SeL and HiSeL as immune enhancers increased the IgG and SIgA antibody produced compared with the mice immunized with *L. brevis* 23017 and vaccine. Some studies have reported that the release of cytokine has a connection with IgG or specific IgG subclass secretion (55). Then, our results showed that both IgG and IL-13 levels increased, possibly due to IL-13 boosts activated B-cell proliferation, differentiation, and the production of IgG (56, 57). We previously mentioned that SIgA production is related to cytokines, which can be easily observed that the increment of IgG and SIgA antibodies was related to promoting cytokine production.

Furthermore, only at 7 days after immunization, the levels of IgG and SIgA of the mice immunized with SeL or HiSeL and vaccine had a better promotion effect than those immunized with *L. brevis* 23017 and vaccine, whereas the mice immunized with *L. brevis* 23017 and vaccine were slightly increased when compared with the mice immunized with vaccine alone, suggesting that SeL and HiSeL induced immune response faster than *L. brevis* 23017, which also shows that SeL and HiSeL effectively made up for the weaknesses of slow IgG antibody production (58) and no SIgA production of alum adjuvant. We speculate that the reason for inducing a more rapid antibody production is that lactic acid bacteria and selenium have a synergistic effect in improving the antibody level (59), and then, they work together to accelerate the production of IgG antibody by B lymphocytes and the increment of SIgA antibody by the intestinal mucosa. However, the specific mechanism of more rapid antibody response is still unknown, so more efforts will be required to explore why antibodies can be induced more quickly.

SeL and HiSeL modulated the Th1 and Th2 immune responses by regulating the release of cytokines. It is very known that cytokines regulate adaptive immunity and innate immunity (60), and their dynamic changes and content reflect the state of immune function. A previous study showed that lactic acid bacteria enhanced Th1 and Th2 immune responses (61). Malyar et al. (62) confirmed that lactic acid bacteria and selenium synergistically promote cytokine secretion and enhance immunity. Se affects the creation of immune cytokines (including IL-1β, IL-2, IL-6, IL-8, IL-10, IL-17, TNF-α, IFN-α, and IFN-γ) and plays an immunomodulatory role by increasing the killing activity and phagocytosis of macrophages and neutrophils (63). Moreover, Se supplementation help increases the number of helpers (CD4) and reduce the number of suppressors (CD8), causing the standardization of immunomodulation (by increasing immune responses index-Th/Tc) (64). Remarkably, Se regulates the phosphorylation level of proteins related to the NF-κB and mitogen-activated protein kinase (MAPK) inflammatory signaling pathways by influencing the expression of selenoprotein S and controlling the release of inflammation-related factors (65). Thus, the prime-boost strategy might effectively enhance Th1 immune responses. SeL and HiSeL improved cellular immune response to a certain extent. In the present work, we detected some cytokines related to SIgA production, including TGF-β1 and Th1 reaction (TNF-α, IFN-γ, and IL-12) and Th2 reaction (IL-4, IL-5, IL-6, IL-10, and IL-13). Previous studies have reported that lactic acid bacteria reduced TNF-α to reduce intestinal epithelium damage (66), which is consistent with our results. We found that the content of TNF-α in mice immunized with SeL or HiSeL and vaccine decreased significantly compared with those immunized with *L. brevis* 23017 and vaccine. In addition, it is reported that lactic acid bacteria increased the cytokines related to Th2 responses (IL-4, IL-5, IL-10, and IL-13) (67), which is consistent with our results. SeL and HiSeL secreted additional cytokines, showing that SeL and HiSeL induced the body to produce Th2 immune responses. Nevertheless, studies have reported that adding lactic acid bacteria improve Th1 response related (IL-12 and IFN-γ) (68), and in our study, only the level of IFN-γ was increased, and the level of IL-12 was decreased. This phenomenon also exists in the mice immunized with SeL or HiSeL and vaccine, which may be due to the excessive secretion of IL-10, inhibiting the secretion of IL-12 (69) and leading to the decline of Th1 immune responses. From this, we inferred that SeL and HiSeL effectively promoted Th2 immune responses and enhanced Th1 immune responses less effectively.

SeL and HiSeL may enhance the immune effect of the vaccine by regulating the oxidative stress state. Numerous studies have found that Se is involved in various biological activities related to immunity and oxidation resistance (70, 71). Antioxidant capacity and oxidative stress tolerance of lactic acid bacteria have been reported (72, 73). Similarly, *L. acidophilus* was reported to improve the antioxidant capacity in rat tissues. Adding *L. acidophilus* reduces the concentration of MDA and increases the concentration of SOD, GSH-Px, and T-AOC, which shows that lactic acid bacteria slow down the oxidative damage of mice after immunization. Studies have also confirmed that nano selenium-enriched lactic acid bacteria have great antioxidant capacity and free radical scavenging efficiency and protect tissues and cells from oxidative damage (74), which is consistent with our results. The concentrations of GSH-Px, SOD, MDA, and T-AOC in the intestine and serum of mice on 1 and 28 days after immunization were detected in the experiment. Compared with the mice immunized with *L. brevis* 23017 and vaccine, MDA in those immunized with SeL or HiSeL and vaccine was further decreased, whereas SOD, GSH-Px, and T-AOC were increased, indicating that SeL and HiSeL have notable antioxidant capacity. There is sufficient evidence to show that optimal Se nutrition combats uncontrolled inflammation, partly because of selenoprotein’s antioxidant and redox-regulating capabilities (75). Se and lactic acid bacteria are inseparable from oxidative stress and the body’s immune function. Thus, they raised body antioxidation to improve the vaccine’s effectiveness.

SeL and HiSeL promoted the immune effect of the vaccine, which was related to their regulation of selenoprotein expression. Animals use Se in the form of selenoprotein, which contains a selenocysteine (SeCys) as their primary Se-containing component (76, 77). Selenoprotein exists in numerous organelles and cells and shows solitary distributions and sensitivity to changes in the level of Se (78). It has been proved that dietary Se affects the composition of the intestinal flora and gastrointestinal colonization, consequently affecting the host Se status and the expression of the Se proteome (79). A previous study found that GPx1 increased in pig spleen by adding selenium to the diet (80). Jin et al. (81) show that Se supplementary significantly increased GPx1 and GPx4 mRNA levels in the liver. Raising the expression of SelN, SelT, and SelW influenced uterine smooth muscle cells after increased Se concentration in food (82). Additional studies demonstrated that increased Se concentration within a certain range upregulated the selenoproteins, including Sephs2, GPx1, GPx2, GPx3, SelW, and SelH (83), which is consistent with our study. In this study, we tested the effects of *L. brevis* 23017, SeL, and HiSeL on the content of selenoprotein. On the first day after immunization, we found that the expression of selenoprotein in mice immunized with *L. brevis* 23017 did not change compared with those immunized with vaccine alone, and then, the expression of GPx1, GPx2, SelH, SephS2, and SelW in mice treated with SeL was remarkably increased, and GPx2 and GPx3 genes in mice treated with HiSeL also increased significantly. However, on the 28th day after immunization, only GPx2, GPx3, and SelW genes were detected to have high expression in mice immunized with SeL and vaccine because selenoproteins play a role in the initial stages of immunity, and then, the effect of selenoprotein decreases gradually over time. Our data and many other studies on Se and immunity provide clear evidence that the GPx2, GPx3, and SelW genes merit special consideration. Some studies have indicated that selenoprotein improves the body’s immunity by regulating various signal pathways (84, 85). Therefore, SeL and HiSeL may improve vaccine effectiveness by increasing the mRNA expression of different selenoproteins. For future studies, the more detailed mechanism of selenoprotein participating in the immunological enhancement process needs further investigation.

In our study, there was a difference in improving the immune effect of the vaccine between SeL and HiSeL. SeL and HiSeL induced a more rapid antibody production and elicited higher IgG and SIgA antibody titers. HiSeL shows some strengths in humoral immune responses, the level of specific IgG antibodies of mice in the HiSeL + Vac group was higher than that in the SeL + Vac group on the seventh, 10th, 14th, and 28th days after immunization. Then, SeL possesses certain strengths in mucosal immune responses, and the total intestinal mucus SIgA antibody level of mice in the SeL + Vac group was higher than that in the HiSeL + Vac group on the 28th day after immunization. In addition, HiSeL stimulates the body to produce more Th2-type cytokines (IL-4, IL-10, and IL-13) on the first day after immunization. However, on the 28th day after immunization, the cytokine levels of the HiSeL + Vac group were lower than that in the SeL + Vac group. In terms of oxidative stress state and selenoprotein expression, SeL revealed more pronounced effects when compared with HiSeL.

In addition, after the challenge, the mortality rate of the mice immunized with SeL and vaccine can reach 0%, which showed SeL significantly improved the vaccine’s protective efficacy against α toxin. Interestingly, the specific IgG antibodies of mice in the HiSeL + 1/2Vac group and the HiSeL + 1/4Vac group were lower than that in SeL + 1/2Vac and SeL + 1/4Vac groups because different immunization doses will affect the HiSeL effect. It is noteworthy that there were no statistical differences between the SeL + Vac group and the HiSeL + Vac group in these indices except for the level of total intestinal mucus SIgA antibody. On the basis of these results, we found that SeL increased the vaccine effect more comprehensively and better than HiSeL, and HiSeL preferred to enhance humoral immunity and Th2-type cytokines secretion. The advantage of SeL in immune enhancement may be attributable to its own secretion of some active substances that create a synergistic effect with selenium for a better and longer-lasting effect on the body, whereas the advantage of HiSeL in humoral immunity may be due to the teichoic acid, peptidoglycan, and metabolites contained in the cell wall after cell inactivation can well action on macrophages and B lymphocytes.

Compared with physical (86) and chemical (87) methods, the recently developed biosynthesis of nano selenium-enriched lactic acid bacteria, the so-called green synthesis employing bacteria, is considered to be a novel, clean, safe, non-toxic, low-cost, and eco-friendly technique (74, 88, 89). The present study shows that SeL and HiSeL may be as safe and effective vaccine immune enhancers. Of note, we successfully explored the new use of nano selenium-enriched lactic acid bacteria, which could be used as promising vaccine immune enhancers to boost immune responses of the vaccine. Then, we also provide new ideas and research directions for applying nano selenium-enriched lactic acid bacteria. However, our study has a few limitations. We cannot determine the appropriate concentration and dose of SeL and HiSeL, low concentration and dose are likely unable to produce protective effects, and high concentration and dose may generate toxic effects for the body. Therefore, determining the appropriate concentration and dose of SeL and HiSeL should significantly improve the vaccine’s protective effect. Next, we will explore optimum concentration and dose range and further analyze the concrete mechanism of Se-selenoprotein in enhancing the immune effect of vaccine by lactic acid bacteria.

## Data availability statement

The original contributions presented in the study are included in the article/**Supplementary Material**. Further inquiries can be directed to the corresponding authors.

## Ethics statement

The Ethical Committee of the Institute approved all scientific experiments. All applicable international and national guidelines for the care and use of animals in experiments were followed. Approval (NEAUEC20210326, 30 March 2021) was obtained from the Institutional Committee of Northeast Agricultural University for animal experiments.

## Author contributions

JG and FW: conceived and designed the experiments. RL, JG, WS, and TS: performed the experiments. FW, HY, and RL: analyzed the data. YN, ZZ, WZ, and KX: contributed reagents, materials, and analysis tools. JG, FW, and RL: wrote the paper. All authors contributed to the article and approved the submitted version.
