# Supplementary figures and images for: Nano selenium-enriched probiotic Lactobacillus enhances alum adjuvanticity and promotes antigen-specific systemic and mucosal immunity
Source: Front Immunol. 2023 Jan 27;14:1116223. doi: 10.3389/fimmu.2023.1116223 (PMC9922588; doi:10.3389/fimmu.2023.1116223)

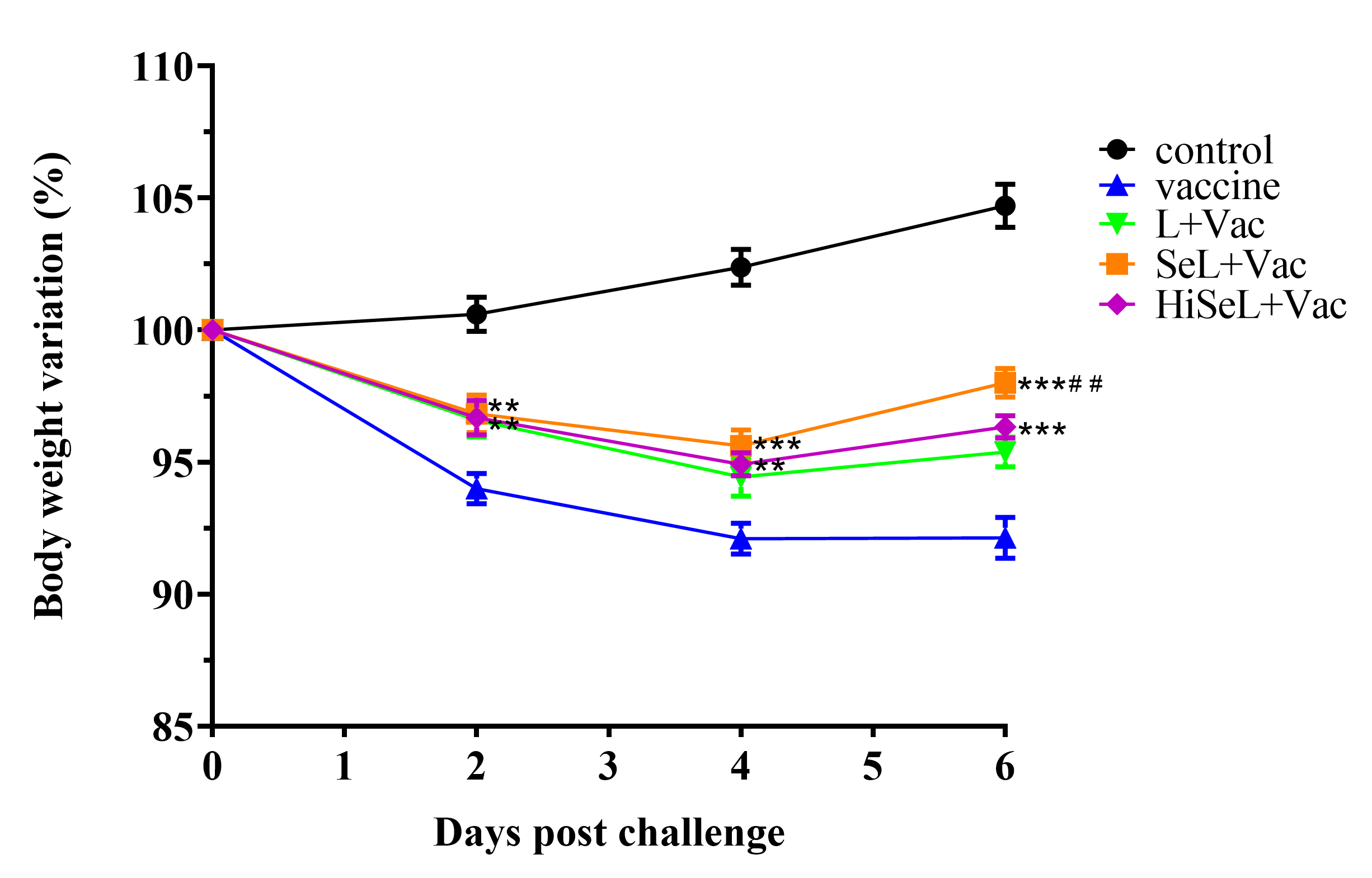

Supplement: Supplementary file 1 [file Image_1.tif]
