# Supplementary material for: Nano selenium-enriched probiotic Lactobacillus enhances alum adjuvanticity and promotes antigen-specific systemic and mucosal immunity
Source: Front Immunol. 2023 Jan 27;14:1116223. doi: 10.3389/fimmu.2023.1116223 (PMC9922588; doi:10.3389/fimmu.2023.1116223)
Supplement: Supplementary file 2 [file Table_1.doc]

**Table S1 |** Primer sequences.

| **Gene** | **Primer** | **Sequence (5’-3’)** | **Reference** |
| --- | --- | --- | --- |
| Hprt1 | Hprt1-F | GCAGTCCCAGCGTCGTG | (28) |
|  | Hprt1-R | GGCCTCCCATCTCCTTCAT |  |
| GPx1 | GPx1-F | GAAGAGATTCTGAATTCCCTCAA | (28) |
|  | GPx1-R | CACACCAGGAGAATGGCAAGA |  |
| GPx2 | GPx-2F | GTGCTGATTGAGAATGTGGC | (28) |
|  | GPx2-R | AGGATGCTCGTTCTGCCCA |  |
| GPx3 | GPx3-F | CCATTTGGCTTGGTCATTCTGGG | (28) |
|  | GPx3-R | CACCTGGTCGAACATACTTGAGAC |  |
| GPx4 | GPx4-F | GCTGGGAAATGCCATCAAATGGA | (28) |
|  | GPx4-R | ACGGCAGGTCCTTCTCTATCAC |  |
| GPx7 | GPx7-F | AGGGGCTCGGTGTCGTTAG | (29) |
|  | GPx7-R | ATGGGAATGCCAGCACATTG |  |
| Txnrd1 | Txnrd1-F | TGACCTCATTGTCATCGGTGG | (29) |
|  | Txnrd1-R | TTCCTCCAAGACCCCACGAA |  |
| Txnrd2 | Txnrd2-F | TTGGGCACAGAAGAAACGGA | (29) |
|  | Txnrd2-R | GGACGGCCCTCTGTTATGTC |  |
| Txnrd3 | Txnrd3-F | ACTCCTTCGAGGCTTTGACC | (29) |
|  | Txnrd3-R | CCAGCCTTTCCACCTGAGTAG |  |
| SelH | SelH-F | CCTTATTCCACCAACGCGCCA | (28) |
|  | SelH-R | GCGTCAGCTCGTACAATGCTC |  |
| SelI | SelI-F | ACTGGTTACTGCTTCCTCTCCTC | (28) |
|  | SelI-R | CTGCTTCACCACTTGTACGCC |  |
| SelK | SelK-F | ATGGAAGAGGGCCACCAGGA | (28) |
|  | SelK-R | TTACCTTCCTCATCCACCAGCC |  |
| SelO | SelO-F | TGACACTGAGTTCCAAAGGCAC | (28) |
|  | SelO-R | GTTAGTGAAGTCAGCACCAGTCAG |  |
| SelT | SelT-F | CTTTAAATGATGTGCCAGTGTGGT | (28) |
|  | SelT-R | GGTAGGGCTATGATCGATGATGTG |  |
| SelW | SelW-F | ATGCCTGGACATTTGTGGCGA | (28) |
|  | SelW-F | GCAGCTTTGATGGCGGTCAC |  |
| SelP | SelP-F | CTCATCTATGACAGATGTGGCCGT | (28) |
|  | SelP-R | AAGACTCGTGAGATTGCAGTTTCC |  |
| SelF | SelF-F | TGCTGCCAAGAAGAAGCTCA | (29) |
|  | SelF-R | CTCCTGACAAAAGCCTGGACT |  |
| SelM | SelM-F | CCGCTGTACCATAACCTGGAG | (29) |
|  | SelM-R | AGTCTCCTTGCGGTAGAAGC |  |
| SelN | SelN-F | TTCTGAGCATGTTCCACCCG | (29) |
|  | SelN-R | CAGCATGGATCCTGAACGCTA |  |
| SelS | SelS-F | TTGCGAGGAGGTGGCTATAA | (29) |
|  | SelS-R | CATGCAAACTGTAGGCAGGC |  |
| SelU | SelU-F | TGATCATGGCTGTGCGAAGA | (29) |
|  | SelU-R | AATCCTCCACTTCGGTCCCT |  |
| Sephs2 | Sephs2-F | CAAGTACGGAGAGGGTCACCA | (28) |
|  | Sephs2-R | CGTTGGAATTATCAGGAGCAGCAG |  |
| Sep15 | Sep15-F | GTTTCAAGCGGCGTCTGCTC | (28) |
|  | Sep15-R | TGCTTCTTCCTGACAGCACCC |  |
| Dio1 | Dio1-F | GGGATTTCATTCAAGGCAGCAGG | (28) |
|  | Dio1-R | TGTGGAGGCAAAGTCATCTACGA |  |
| Dio2 | Dio2-F | ATTTGCTGATCACGCTTCAG | (29) |
|  | Dio2-R | GCTCAGAAACAGCACCATGT |  |
| Dio3 | Dio3-F | CCAGCCACGCTCTGTCAATA | (29) |
|  | Dio3-R | ATCCCGAAGGAAGAGAGCCT |  |
| β-actin | β-actin-F | CCAGTTGGTAACAATGCCATGT | (30) |
|  | β-actin-R | GGCTGTATTCCCCTCCATCG |  |
| IL-4 | IL-4F | GCCGATGATCTCTCTCAAGTGA | (30) |
|  | IL-4R | GGTCTCAACCCCCAGCTAGT |  |
| IL-10 | IL-10F | CGCAGCTCTAGGAGCATGTG | (30) |
|  | IL-10R | GCTCTTACTGACTGGCATGAG |  |
| IL-12 | IL-12F | ACAGCACCAGCTTCTTCATCAG | (31) |
|  | IL-12R | TCTTCAAAGGCTTCATCTGCAA |  |
| IFN-γ | IFN-γF | AGACAATCAGGCCATCAGCA | (31) |
|  | IFN-γR | TGGACCTGTGGGTTGTTGAC |  |
