# Supplementary material for: Nano selenium-enriched probiotic Lactobacillus enhances alum adjuvanticity and promotes antigen-specific systemic and mucosal immunity
Source: Front Immunol. 2023 Jan 27;14:1116223. doi: 10.3389/fimmu.2023.1116223 (PMC9922588; doi:10.3389/fimmu.2023.1116223)
Supplement: Supplementary file 3 [file Table_2.doc]

**Table S2 |** Challenge protection results of α toxin of *Clostridium perfringens* type A.

| **Group** | **The number of diseased mice** | **Morbidity rate** | **The number of dead mice** | **Mortality rate** |
| --- | --- | --- | --- | --- |
| control | 0/5 | 0% | 0/5 | 0% |
| vaccine | 4/5 | 80% | 0/5 | 0% |
| 1/2 vaccine | 5/5 | 100% | 2/5 | 40% |
| 1/4 vaccine | 5/5 | 100% | 3/5 | 60% |
| SeL+Vac | 0/5 | 0% | 0/5 | 0% |
| SeL+1/2 Vac | 1/5 | 20% | 0/5 | 0% |
| SeL+1/4 Vac | 1/5 | 20% | 0/5 | 0% |
| HiSeL+Vac | 0/5 | 0% | 0/5 | 0% |
| HiSeL+1/2 Vac | 5/5 | 100% | 1/5 | 20% |
| HiSeL+1/4 Vac | 5/5 | 100% | 3/5 | 60% |
