# Supplementary material for: Nano selenium-enriched probiotic Lactobacillus enhances alum adjuvanticity and promotes antigen-specific systemic and mucosal immunity
Source: Front Immunol. 2023 Jan 27;14:1116223. doi: 10.3389/fimmu.2023.1116223 (PMC9922588; doi:10.3389/fimmu.2023.1116223)
Supplement: Supplementary file 4 [file Table_3.doc]

**Table S3 |** Survival rate of mice in α toxin of *Clostridium perfringens* type A neutralization assay.

| **Group** | **Corresponding days of rabbit serum after immunization** | | | | | |
| --- | --- | --- | --- | --- | --- | --- |
|  | **7 d** | | **10 d** | | **14 d** | |
| control | | 0% | | 0% | | 0% |
| vaccine | | 0% | | 66% | | 100% |
| SeL+Vac | | 0% | | 100% | | 100% |
